# Development of a RPA-CRISPR-Cas12a Assay for Rapid, Simple, and Sensitive Detection of *Mycoplasma hominis*

**DOI:** 10.3389/fmicb.2022.842415

**Published:** 2022-04-08

**Authors:** Jialing Chen, Yinger Huang, Bin Xiao, Hao Deng, Kunxiang Gong, Kun Li, Linhai Li, Wenbo Hao

**Affiliations:** ^1^Institute of Antibody Engineering, School of Laboratory Medicine and Biotechnology, Southern Medical University, Guangzhou, China; ^2^Key Laboratory of Antibody Engineering of Guangdong Higher Education Institutes, Southern Medical University, Guangzhou, China; ^3^Department of Laboratory Medicine, The Sixth Affiliated Hospital of Guangzhou Medical University, Qingyuan People’s Hospital, Qingyuan, China; ^4^Stem Cell Clinical Transformation and Application Center, Shenzhen Qianhai Shekou Free Trade Zone Hospital, Shenzhen, China; ^5^Department of Gynecology and Obstetrics, Guangzhou Women and Children’s Medical Center, Guangzhou, China; ^6^Guangdong Provincial Key Laboratory of Construction and Detection in Tissue Engineering, Southern Medical University, Guangzhou, China

**Keywords:** lateral flow strip, point-of-care diagnosis, CRISPR-Cas12a, *Mycoplasma hominis*, recombinase polymerase amplification

## Abstract

*Mycoplasma hominis*, which is difficult to culture and identify by ordinary methods, is one of the smallest pathogens in the human genitourinary tract causing urogenital infections. A CRISPR-Cas12a-based detection system might provide a novel application for *M. hominis* nucleic acid detection in molecular diagnostics. A plasmid containing the glyceraldehyde-3-phosphate dehydrogenase gene of *M. hominis* (ATCC_27545) as the positive control was constructed by homologous recombination. The active Cas12a protein was purified by affinity chromatography. The primers for recombinase polymerase amplification (RPA), the CRISPR RNA (crRNA), and the ratio of Cas12a to crRNA were further optimized. Finally, the sensitivity, specificity, and clinical effectiveness of the Cas12a detection system were confirmed. We successfully constructed and optimized a novel nucleic acid detection system for *M. hominis* based on RPA-CRISPR-Cas12a, and the whole process takes only 1 h. The limit of detection for the gap gene of *M. hominis* was 3 copies/μl and no cross-reactivity with other urogenital pathogens appeared. In the evaluation of 111 clinical samples, the sensitivity and specificity were both 1.000 and the area under the curve of the receiver operating characteristic was 1.000 (*p* < 0.001), indicating that the RPA-Cas12a-fluorescent assay was fully comparable to the traditional culture method. Finally, the RPA-Cas12a detection system can also be combined with lateral flow strips (LFS) to achieve visual detection. We successfully developed a low-cost and rapid detection method of *M. hominis* based on RPA-Cas12a technology. This method realized by fluorescence value readout and visual detection by LFS could be applied in population screening and resource-limited conditions.

## Graphical Abstract

Schematic of *Mycoplasma hominis* nucleic acid detection based on the CRISPR-Cas12a system. Clinical samples from cervical or urethral swabs were collected and incubated with lysis buffer to release nucleic acid (10 min). Extracted DNA (1 μl) is subjected to the RPA reaction with specific primers at 37°C. After 20 min, RPA product was subjected to the CRISPR-Cas12a reaction for cleavage. The collateral nuclease activity of Cas12a (250 nM) proteins were activated upon specific binding to crRNA (crRNA3, 62.5 nM) and the DNA product; thus, Cas12a cut the quenched fluorescent ssDNA reporter (125 nM) (30 min). The generated fluorescence signal would be measured by a fluorescence plate reader or visualized by lateral flow strips.

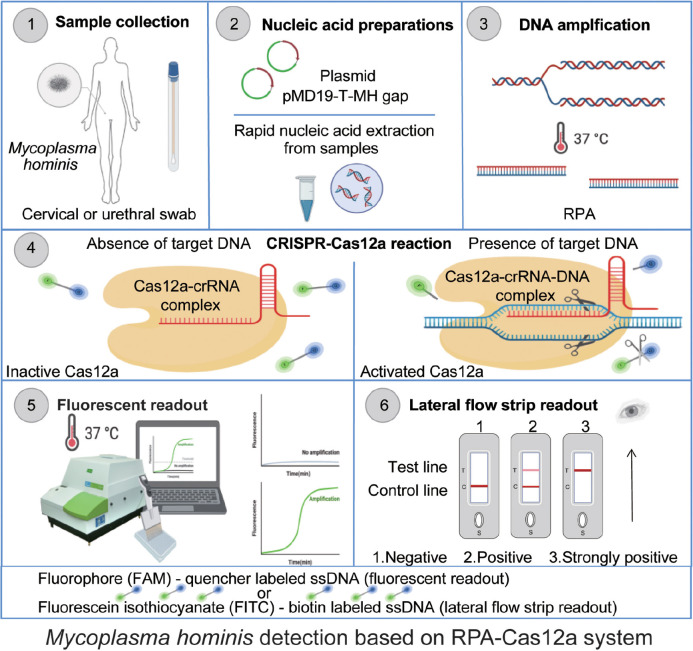

## Introduction

The bacterium *Mycoplasma hominis*, an obligatory parasite in human genitourinary tract, belongs to the class Mollicutes (Margarita et al., 2020). It is characterized by the absence of a rigid cell wall and has one of the smallest genomes among self-replicating organisms. In addition to causing human urogenital infection (Wang H. et al., 2021), it can also induce systemic infection in immunocompromised individuals, including those with primary or medically induced immunodeficiencies (Sampath et al., 2017). Recent studies have shown that *M. hominis* infection is associated with premature birth, malformation, and even death of the fetus, resulting in seriously adverse consequences for pregnant women and babies (Michou et al., 2014; Kersin et al., 2020; Ansari et al., 2021; Paira et al., 2021). Hence, the early detection of the infection may reduce maternal and neonatal morbidity (Ray et al., 2015).

Due to the lack of peptidoglycan cell wall, *M. hominis* cannot be detected with Gram staining. Therefore, current diagnostic methods of *M. hominis* mainly rely on laboratory-based tests including liquid medium culture (Keskin et al., 2018) and quantitative real-time polymerase chain reaction (qRT-PCR) (Patel and Nyirjesy, 2010; Campos et al., 2015; Ozturk et al., 2019). The culture of *M. hominis* is the gold standard diagnostic method. However, it is difficult to culture *M. hominis* on the traditional growth medium. The isolation and culture of *mycoplasma* require cholesterol and nucleic acid precursors in the culture medium. Compared with other common pathogens in the reproductive tract, it has a relatively slow growth rate, which requires a culturing process of 48 to 96 h (Cunningham et al., 2013), making the detection labor-intensive and time-consuming with low specificity (Ferandon et al., 2011). Also, qRT-PCR requires complex instruments and technical expertise, which restrict their use to centralized laboratories (Qi et al., 2019). In view of the above characteristics, it is necessary to establish a rapid, low-cost, sensitive, and convenient laboratory diagnosis method as a novel alternative.

Recently, a clustered regularly interspaced short palindromic repeats (CRISPR)-associated nuclease system has provided potential applications for rapid and sensitive molecular diagnosis (Gootenberg et al., 2018). The CRISPR-Cas system consists of a genetic locus containing palindromic repeats, non-repetitive, unique spacer sequences, and 6–20 adjacent genes encoding Cas proteins. CRISPR-Cas12a (also called Cpf1) is an RNA-guided DNA endonuclease that cleaves target double-stranded DNA (dsDNA) and referring to non-specific cleavage of single-stranded DNA (ssDNA) by an “activated” ternary Cas12a–crRNA–DNA duplex with several distinct advantages in comparison with Cas9, such as lower mismatch tolerance and greater specificity (Gootenberg et al., 2018). However, Cas12a on its own is theoretically not sensitive enough to detect low levels of nucleic acids. To improve its detection sensitivity, CRISPR-Cas12a detection is typically combined with an isothermal amplification step, such as recombinase polymerase amplification (RPA) (Kanitchinda et al., 2020). RPA is reliable and considered as one of the nucleic acid amplification technologies for molecular diagnostics, which enables both sensitive and rapid isothermal DNA amplification at a temperature range of 37–42 °C (Kanitchinda et al., 2020). Combining the collateral effect of Cas12a with RPA and a fluorescence readout, a detection platform called DNA endonuclease targeted CRISPR trans reporter (DETECTR) (Chen et al., 2018) has been recently devised and proved to be applicable to nucleic acid detection of pathogens, with higher sensitivity.

By specifically and accurately cutting dsDNA matched to its corresponding specific crRNA with a thymine nucleotide-rich protospacer-adjacent motif (PAM) sequence, Cas12a attains the capability to further cut ssDNA probes labeled with fluorescent tag or biotin (Dai et al., 2019; Wang et al., 2020). Readout by fluorescence values is measured by a fluorescence reader or displayed by lateral flow strips, and the cleavage effect represents the presence of target nucleic acid in the original sample (Chen et al., 2018).

Cas12a is rarely used in the detection of urogenital pathogens, and there is no relevant report in the diagnosis of *M. hominis*. Here, we provided the application of CRISPR-Cas12a detection in simple, rapid, sensitive, and reliable *M. hominis* diagnosis. Different RPA primers and crRNAs were tested for their efficiency, and the optimized RPA-Cas12a workflow was then evaluated for sensitivity and specificity. Visualized detection was also assessed for its compatibility with the assay. Finally, the performance of the RPA-Cas12a system was compared with the culturing method in the detection of *M. hominis* in clinical samples. The reaction process of Cas12a-based *M. hominis* DNA detection system is shown in the Graphical abstract.

## Materials and Methods

### Reagents and Instruments

Primers for RPA were designed using NCBI Primer-BLAST^1^, and *in vitro* transcribed (IVT) templates for crRNA were ordered from Genewiz (Suzhou, China). Commercial Cas9, Cas12a, Cas13a, and FAM-TTTTT-Quencher used in fluorescent reporter assay and FITC-TTTTTT-Biotin probe used in gold nanoparticle-labeled lateral flow strips were obtained from Biolifesci (Guangzhou, China). The detailed sequences are listed in Supplementary Table 2. *M. hominis* genomic DNA was extracted using lysis buffer for Microorganism to Direct PCR (Takara, Tokyo, Japan). The TwistAmp^®^ Basic kit for RPA test was purchased from TwistDx (Cambridge, United Kingdom). T7 RNA polymerase, the NTP mix, and the RNA purification kit were purchased from New England Biolabs (Ipswich, MA, United Kingdom). RNase-free water and Recombinant DNase I (RNase-free) were purchased from Takara. RNase-free water was used in all experiments. Streptavidin was purchased from Solarbio (Beijing, China), while Goat anti-Rabbit IgG and gold-nanoparticles–Rabbit anti-FITC were purchased from Bioss (Beijing, China). The fluorescence quantifications were measured with a Wallac 1420 plate reader (PerkinElmer, United States). Amplification was confirmed by 3% agarose gel electrophoresis and the RPA products were visualized using a Gel Doc system (Bio-Rad, United States). The relevant reagents are presented in Supplementary Table 1.

### Clinical Specimens and Ethics Statement

Specimens obtained for testing included cervical or vaginal swabs for women and urethral swabs for men. A total of 178 samples were collected from sexually transmitted disease (STD) clinics at Shantou Second People’s Hospital in China from June 2020 to November 2020, including 29 samples from men and 149 samples from women. We collected two swabs from the same part of a patient, one for culture and one for nuclear acid detection with the RPA-CRISPR-Cas12a assay. Samples were processed within 2 h after collection. The study was approved by the Institutional Medical Ethics Review Board of Shantou Second People’s Hospital.

### Nucleic Acid Preparation

Clinical samples of swab heads were resuspended in phosphate buffered saline (PBS, pH 7.4) and centrifuged at 14,000 × *g* for 1 min. Bacterial cells were harvested by centrifugation. After removing the supernatant, 50 μl of lysis buffer for Microorganism to Direct PCR was added to the precipitates and heated for 10 min at 85°C according to the manufacturer’s instructions. The supernatant extracts were directly used for the RPA reactions of the *M. hominis*. A 280-bp fragment of the glyceraldehyde-3-phosphate dehydrogenase (gap) gene of *M. hominis* (Supplementary Table 2) was amplified and further cloned into a pMD19-T vector (Supplementary Table 3) according to the manufacturer’s instructions (Solarbio, Beijing, China) to construct the positive control called “pMD19-T-MH gap.” The successful construction of the pMD19-T-MH gap was confirmed by DNA sequencing (Genewiz. Suzhou, China). The pMD19-T-MH gap carrying target genes was extracted with a plasmid extraction kit (Omega, United States) and quantified with a nucleic acid analyzer (Bibby Scientific Limited, United Kingdom). *M. hominis* plasmid was used as a positive control to establish Cas12a detection assay in this study.

### Optimization of the Recombinase Polymerase Amplification Reaction System

In order to get a satisfactory performance of the RPA reaction system, several parameters were supposed to be optimized, such as the concentration of the primers, reaction temperature, and incubation time. The concentration of primers was first optimized. We added 0.1, 0.25, 0.5, and 1 μM primers to the RPA reaction system, respectively (RPA reaction system can be found in Supplementary Table 6). After 20 min of reaction, the amplified products were observed by agarose electrophoresis. What is more, the amount of amplified products could be influenced by the reaction temperature and incubation time. The reactions were incubated at five temperatures (37, 38, 39, 40, and 42°C) and five incubation times (5, 10, 20, 30, and 40 min). After reaction, the amplified products were observed by agarose electrophoresis.

### AsCas12a Protein Expression and Purification

The Cas12a nuclease derived from *Acidaminococcus* sp. (AsCas12a, formerly AsCpf1) was obtained from the 6His-MBP-TEV-huAsCpf1 (AsCas12a) expression vector of Sail Health Biology. The fusion protein contained an N-terminal His6-MBP tag followed by a tobacco etch virus (rTEV) protease cleavage site. The AsCas12a protein was expressed in *E. coli Rosetta2* (*DE3*) that were induced with 0.1 mM isopropyl-1-thio-β-D-galactopyranoside (IPTG, sigma) at OD_600_ = 0.6 for 16 h at 21°C in LB medium, supplemented with 34 μg/ml ampicillin. Cells were collected and lysed by sonication (1 s on and 2 s off) in buffer containing 20 mM Tris–HCl, pH 7.5, 1 M NaCl, 20 mM imidazole, and 10% glycerol. After centrifugation, the supernatant was incubated with nickel-nitrilotriacetic (Ni-NTA) resin (HisPurTN Ni-NTA resin, ThermoFisher Scientific). The bound protein was eluted with buffer containing 20 mM Tris–HCl, pH 7.5, 150 mM NaCl (with 50, 100, and 250 mM imidazole). The eluted AsCas12a protein was digested with rTEV protease (Solarbio, Beijing, China) at 16°C overnight to remove the His6-MBP tag. We again combined the mixture with Ni-NTA. The miscellaneous proteins with His-tag (including Cas12a with MBP-tag and other miscellaneous proteins) would be combined with Ni NTA while the pure Cas12a was not combined with Ni NTA. After elution, the rest was the purified Cas12a. Furthermore, the purified Cas12a was concentrated to a concentration of 3 mg/ml on Amicon Ultra-4 Centrifugal Filter Unit (Merck-Millipore), with buffer containing 20 mM Tris–HCl, pH 7.5, 1 M NaCl, and 10% glycerol as eluent.

### *In vitro* Transcription and Purification of crRNA

Based on previous literature, the multiple sequence alignment results showed the 16S rRNA and gap gene have high conservation among all the sequences analyzed (Daher et al., 2016; Keskin et al., 2018). Therefore, RPA primers were designed based on the conserved sequences. In advance, the crRNAs were designed for the amplified RPA products with CRISPR-DT tool^2^ and they were generated from a DNA template, which is the reverse complement of target crRNA sequences *via in vitro* transcription (Supplementary Table 4). Since we used T7 transcription, an additional T7 RNA polymerase promoter was placed forward to the crRNA sequences to allow T7 transcription (Supplementary Table 5). crRNA sequences and corresponding *in vitro* transcriptional DNA templates for producing crRNA are displayed in Supplementary Table 2.

### RPA-CRISPR-Cas12a-Based Fluorescent (RPA-Cas12a-Fluo) Assay

Genomic DNA extract (1 μl) was used as input for RPA reaction (37°C, 20 min). The complete detection system of RPA-Cas12a-Fluo assays was performed with 250 nM purified AsCas12a, 62.5 nM crRNA3, 1 μl target DNA (the amplified RPA products), and 125 nM quenched fluorescent ssDNA reporter mixed in Cas12a reaction buffer (40 mM Tris–HCl, 60 mM NaCl, and 6 mM MgCl_2_, pH 7.3) in a 20-μl reaction volume (Supplementary Table 7). The mixture was incubated in a 384-well microplate format (SPL Lifesciences, Pocheon, South Korea) and performed at 37°C for 40 min on a fluorescence plate reader (PerkinElmer WALLAC 1420) (λex: 492 nm; λem: 518 nm). The subsequent reaction systems are based on the above description called “RPA-Cas12a-Fluo assay,” unless otherwise stated.

### Liquid Medium Culture

The culture of *M. hominis* was performed by a *Mycoplasma* ICS Kit (Livzon Group Reagent Factory, Zhuhai, China). The clinical specimens were injected into the liquid medium and placed in the candle jar. After the culturing at 37°C for 24–48 h, the color of the culture tube was observed every day. The medium changing from yellow to pink or red suggests the growth of *M. hominis*.

### Clinical Specimen Evaluation by Liquid Medium Culture and RPA-Cas12a-Fluo Assay

Sixty-seven *M. hominis* negative clinical samples (13 from men and 54 from women) were tested based on the RPA-Cas12a-Fluo assay, and the average value and standard deviation were calculated. The fluorescence threshold of the RPA-Cas12a-Fluo assay was set as the average value plus three times the standard deviation. The result higher than the fluorescence threshold was determined to be positive. Subsequently, 111 clinical specimens (16 from men and 95 from women) were tested based on the RPA-Cas12a-Fluo assay and compared with the results of the liquid medium culture as a gold standard. The results of these two methods were analyzed for correlation and consistency.

### Lateral Flow Detection Reactions

Combining the RPA-CRISPR-Cas12a process with lateral flow strips, we further developed a RPA-CRISPR-Cas12a lateral flow strip assay (RPA-Cas12a-LFS). Similar to that reported in another study (15), the kind of LFS (#JY0209 from Tiosbio, Beijing, China) is different from other conventional test strips. If the sample is positive, the C line and T line will both appear, while the C line will not appear with only the T line present when the sample is strongly positive. The reporter of ssDNA probe was labeled with fluorescein isothiocyanate (FITC) at the 5′ and biotin at 3′, respectively. The RPA-Cas12a-LFS assay was performed with 250 nM purified AsCas12a, 62.5 nM crRNA, 1 μl of target DNA (the amplified RPA products), and 125 nM ssDNA reporter labeled with FITC and Biotin mixed in Cas12a reaction buffer (40 mM Tris–HCl, 60 mM NaCl, and 6 mM MgCl_2_, pH 7.3) in a 20-μl reaction volume. To analyze the results of Cas12a detection with LFS, 20 μl of the above mixture was added to 60 μl of PBS following loading onto the sample pad in LFS for 2 min. To determine the intensity of T lines, we converted the strip image to 8-bit grayscale and measured the gray values of the lines using ImageJ, and calculated the indicated relative T line intensity of clinical samples by subtracting them by the gray value of the negative control.

### Specificity Test of the Proposed Method

Since there are many bacteria present in the urinogenital tract, the swabs are prone to be contaminated with other bacteria during sample collection. To avoid interference by other normal urinogenital bacteria or urinogenital tract pathogens, we selected four normal urinogenital bacteria and six common urinogenital tract pathogens as the specificity evaluation. Eleven pathogens including target pathogen *M. hominis* (ATCC_27545), normal urinogenital bacteria (including *Staphylococcus aureus* (ATCC 10832), *Staphylococcus saprophyticus* (ATCC 15305), *Klebsiella pneumoniae* (ATCC 10031), *Escherichia coli* (ATCC 25922), and other urinogenital tract pathogens [including *Candida albicans* (ATCC 11006), *Shigella flexneri* (ATCC 12022), *Ureaplasma urealyticum* (ATCC 33697), *Chlamydia trachomatis L2* (ATCC VR-902B), *Neisseria gonorrhoeae* (ATCC 49926), and *Mycoplasma genitalium* G37 (ATCC 33530)] were detected to determine the specificity of the RPA-Cas12a Fluo assay and RPA-Cas12a-LFS assay, which were performed as described in Section “RPA-CRISPR-Cas12a-Based Fluorescent (RPA-Cas12a-Fluo) Assay” and Section “Lateral Flow Detection Reactions”, respectively.

### Statistical Analysis

Statistical analyses were performed with GraphPad Prism 7. Each experiment was repeated three times for each sample. The initial fluorescence from all samples and the background condition (no templates) fluorescence were subtracted to generate background subtracted fluorescence. The results are presented as mean ± SD unless otherwise indicated. Regarding the liquid medium culture assay as a gold standard, the sensitivity of the developed assay was calculated as follows: sensitivity = true positive/(true positive + false negative), while the specificity was calculated as follows: specificity = true negative/(true negative + false positive).

## Results

### Construction of Plasmid With *M. hominis*-Specific Sequence and Specific Amplification by Recombinase Polymerase Amplification

**FIGURE 1:**
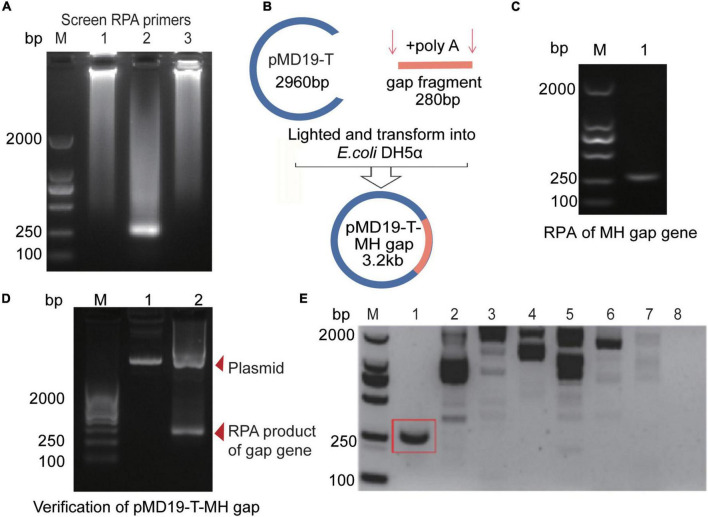
RPA primers optimization and construction of the pMD19-T-MH gap plasmid. **(A)** Optimization of RPA products of *M. hominis* target sequences with three different primers. **(B)** Construction process of recombinant pMD19-T-MH gap with a 280-bp *M. hominis* gap gene fragment. **(C)** Agarose gel electrophoresis for detecting RPA products of the gap gene. **(D)** Agarose gel electrophoresis for detecting the pMD19-T-MH gap (3.2 kb) and the RPA products of the gap gene (280 bp). **(E)** Agarose gel electrophoresis for detecting RPA products with different microorganisms. 1, *M. hominis*; 2, *C. albicans*; 3, *E. coli*; 4, *K. pneumoniae*; 5, *S. aureus*; 6, *S. flexneri*; 7, *S. saprophyticus*; 8, *U. urealyticum*. bp, base pair; M, marker; MH, *M. hominis*; gap, glyceraldehyde-3-phosphate dehydrogenase; RPA, recombinase polymerase amplification.

We first designed three RPA primers (Supplementary Table 2) to amplify *M. hominis*-specific sequence (Supplementary Figure 2). The results showed that the second pair of primers matched to the gap gene locus was ideal for RPA amplification (Figure 1A). The length of the RPA product was 280 bp (Figure 1C). We then inserted the RPA product to the pMD19-T vector to construct the recombinant plasmid called “pMD19-T-MH gap.” The original 280-bp amplified fragment was ligated into the 2.96-kb pMD19-T vector (Figure 1B) by homologous recombination to form a plasmid “pMD19-T-MH gap,” which was 3.24 kb in Lane 1 in Figure 1D. Lane 2 in the figure was the 280-bp product of the RPA amplification (Supplementary Table 6) with the plasmid “pMD19-T-MH gap” with the sequencing result shown in Supplementary Figure 1. The sequencing results further confirmed that the target sequence was correct and could be used as a positive control in subsequent experiments. Furthermore, we explored the specificity of the RPA primers. After extracting DNA from the microorganisms (*M. hominis, C. albicans*, *E. coli*, *K. pneumoniae*, *S. aureus, S. flexneri, S. saprophyticus, U. urealyticum, C. trachomatis, N. gonorrhoeae*, and *M. genitalium*), we added it to RPA amplification at 37°C for 20 min. The results of nucleic acid electrophoresis revealed that our RPA primer 2 were with remarkable specificity, which is conducive to the Cas12a detection system (Figure 1E and Supplementary Figure 4A).

### AsCas12a Protein Expression, Purification, and Verification

**FIGURE 2:**
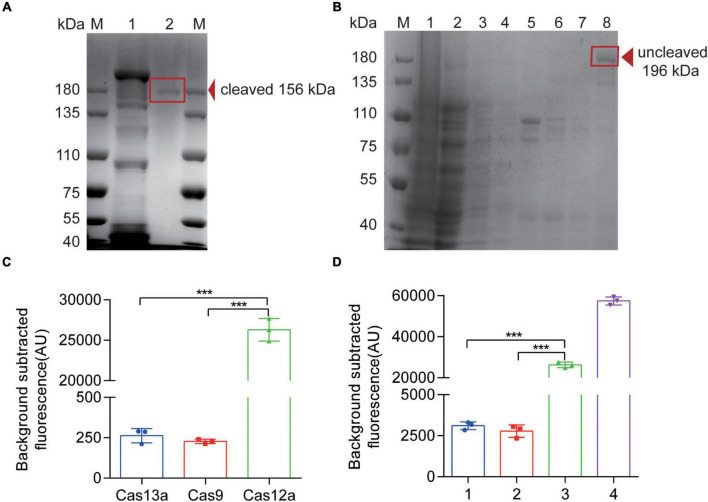
Expression, purification, and verification of Cas12a protein. **(A)** SDS-PAGE of Cas12a protein. M, 180 kDa protein marker; 1, A band above 180 kDa eluted from the Ni-NTA column, consistent with the size of an MBP-AsCas12a fusion (196 kDa); 2, After digestion of the rTEV enzymes, a lower molecular weight band appeared, consistent with the size of 156 kDa, which was the pure AsCas12a. **(B)** SDS-PAGE of Cas12a protein. M, 180 kDa protein marker; 1, bacterial lysate pellet without IPTG induction; 2, bacterial lysis supernatant without IPTG induction; 3, bacterial lysis supernatant with IPTG induction; 4, flow through solution of purification; 5, 0.05 mol/L imidazole eluted proteins with UV value > 0.05; 6, 0.1 mol/L imidazole eluted proteins with UV value > 0.05; 7, 0.25 mol/L imidazole eluted proteins with UV value > 0.05; 8, 0.25 mol/L imidazole eluted proteins after concentration. **(C)** Fluorescence results of Cas12a activity verification compared with Cas13a and Cas9 proteins. **(D)** Fluorescence results of Cas12a activity verification. 1, false crRNA was added to the detection system instead of target crRNA; 2, inactive Cas12a (250 nM) is added to the detection system instead of Cas12a protein; 3, Complete detection system; 4, only DNase and the quenched fluorescent DNA probe reacted in RNase-free H_2_O. The data are presented as the mean ± SD from three independent experiments. ***p < 0.001. M, marker.

In order to establish an effective detection system, it is necessary to obtain Cas12a with high purity and activity. We used a plasmid 6His-MBP-TEV-huAsCpf1 (AsCas12a) containing Cas12a protein-coding sequence for prokaryotic expression and purified it properly with Ni-NTA resin. After elution, Cas12a with MBP-tag (approximately 196 kDa) was collected (Figure 2B). Subsequently, we obtained the purified Cas12a (approximately 156 kDa) *via* rTEV enzyme digestion (Figure 2A). The results of Cas12a expression, purification, and concentration are shown in Figures 2A,B, indicating that we have obtained the purified Cas12a protein. To further confirm the cleavage activity of Cas12a, different compounds were added to the reaction. In Figure 2C, in order to verify the specificity of Cas12a and corresponding crRNA (crRNA3, 62.5 nM), we compared the activity of Cas12a (250 nM) with Cas13a (250 nM) and Cas9 (250 nM). Other reaction components and conditions were consistent with the RPA-Cas12a-Fluo assay (Supplementary Table 7). After 40 min of reaction, the fluorescence value was recorded. In Figure 2D, the reactants of the complete detection system were consistent with the RPA-Cas12a-Fluo assay or replaced by false crRNA (Supplementary Table 2) or inactive Cas12a protein after irradiation by ultraviolet lamp overnight at room temperature. The results showed that only the active Cas12a protein combined with the correct target fragment and specific crRNA3 could have exerted the cleavage and collateral cleavage effect and produced obvious fluorescence values. In advance, we compared the commercially available Cas12a with Cas12a purified by us with the RPA-Cas12a-Fluo assay, and the result is shown in Supplementary Figure 3. It was inspiring that the nuclease activity of the Cas12a purified by ourselves was better.

### Optimization of the CRISPR-Cas12a Detection System

**FIGURE 3:**
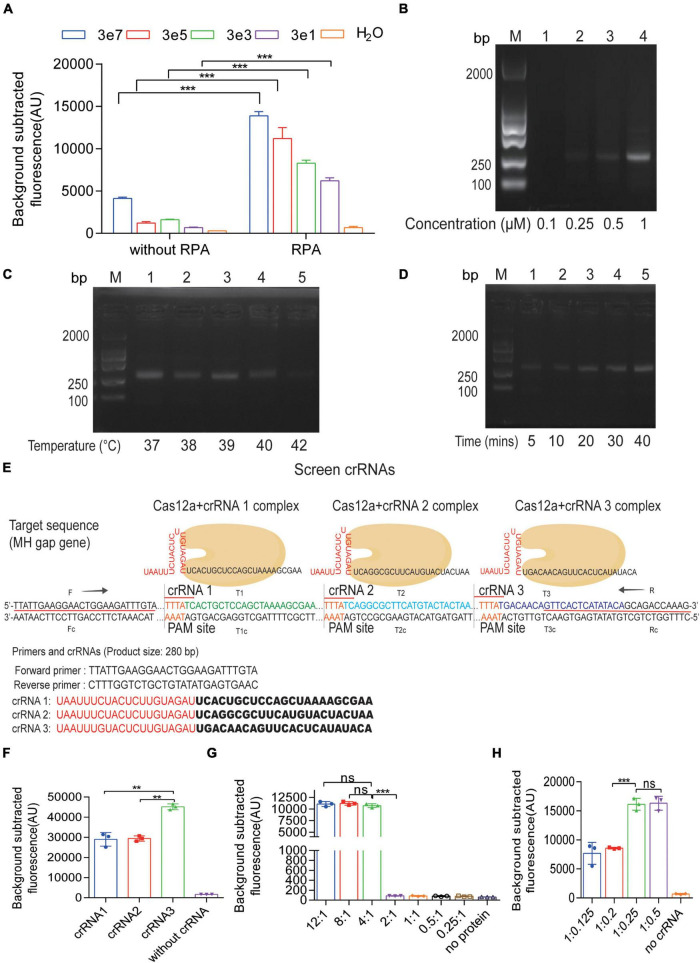
Optimization of the CRISPR-Cas12a fluorescent assay. **(A)** Detection of fluorescent signals from serial 10-fold dilutions of the pMD19-T-MH gap by CRISPR-Cas12a fluorescent assay with or without RPA amplification. **(B)** Agarose gel electrophoresis results of PRA products amplified by different concentrations of primers. **(C)** Agarose gel electrophoresis results of PRA products amplified by different temperature. **(D)** Agarose gel electrophoresis results of PRA products amplified by different time. **(E)** The sequences of RPA primers and three crRNAs for the gap gene of *M. hominis*. **(F)** Fluorescent results of the pMD19-T-MH gap detected by the RPA-Cas12a-Fluo assay with crRNA1, crRNA2, and crRNA3. **(G)** Fluorescent results of the pMD19-T-MH gap detected by the RPA-Cas12a-Fluo assay with a different Cas12a:crRNA ratio. The ratio of 4:1 reached the highest fluorescent value. **(H)** Fluorescent results of the pMD19-T-MH gap detected by the RPA-Cas12a-Fluo assay with a different Cas12a:crRNA ratio. The ratio of 1:0.25 reached the highest fluorescent value. The data are presented as the mean ± SD from three independent experiments. **p < 0.01; ***p < 0.001; ns, *p* > 0.05. Abbreviations: MH, *M. hominis*; RPA, recombinase polymerase amplification; bp, base pair; M, marker; gap, glyceraldehyde-3-phosphate dehydrogenase.

To improve the efficiency of Cas12a detection, we have to optimize the reaction system. According to Figure 3A, without RPA procedure, the sensitivity of Cas12a detection could only just reach 3 × 10^7^ copies per reaction. However, the combination of CRISPR-Cas12a and RPA, called the RPA-Cas12a-Fluo assay, achieved a notable limit of detection (LOD) of three copies of *M. hominis* DNA. Next, to evaluate the effect of primer concentrations in RPA, we added different concentrations of primers in RPA reaction (Figure 3B). In order to save the cost, we chose 0.5 μM of primers instead of 1 μM. Although it seems that 0.25 μM of primers produced a weak band, in order to obtain more RPA products, we finally chose the 0.5 μM of primers as the optimal reaction condition. Also, we optimized the reaction temperature and time of RPA. Using gradient temperature, duration in the reaction and a constant amount of pMD19-T-MH gap template, we found that the reaction at 37°C for 20 min produced the brightest band of the target product (Figures 3C,D). As CRISPR-Cas12a recognizes a PAM, the crRNAs may enable Cas12a to specifically detect *M. hominis* DNA. Three crRNAs (Supplementary Table 2) with PAM (TTTN) targeted the gap gene of *M. hominis.* We first designed and evaluated different crRNAs (crRNA1, crRNA2, and crRNA3) with the RPA-Cas12a-Fluo assay. As shown in Figures 3E,F, all three crRNAs had the ability to react with Cas12a, which caused the increase of fluorescence value compared with the control group. Among them, the fluorescence value of the crRNA3 group was the highest. Hence, we selected crRNA3 for *M. hominis* detection. Additionally, the ratio of Cas12a to crRNA was also optimized. AsCas12a:crRNA3 was set with varying proportions in the RPA-Cas12a-Fluo assay, and the fluorescence signals were collected. The subtracted fluorescence showed that a 4:1 ratio of Cas12a:crRNA3 (250 nM purified AsCas12a:62.5 nM crRNA3) ensured Cas12a-mediated cleavage with less amount of reactants consumed (Figures 3G,H).

### Construction of the RPA-Cas12a-Fluo Assay and Validation of Clinical Samples

**FIGURE 4:**
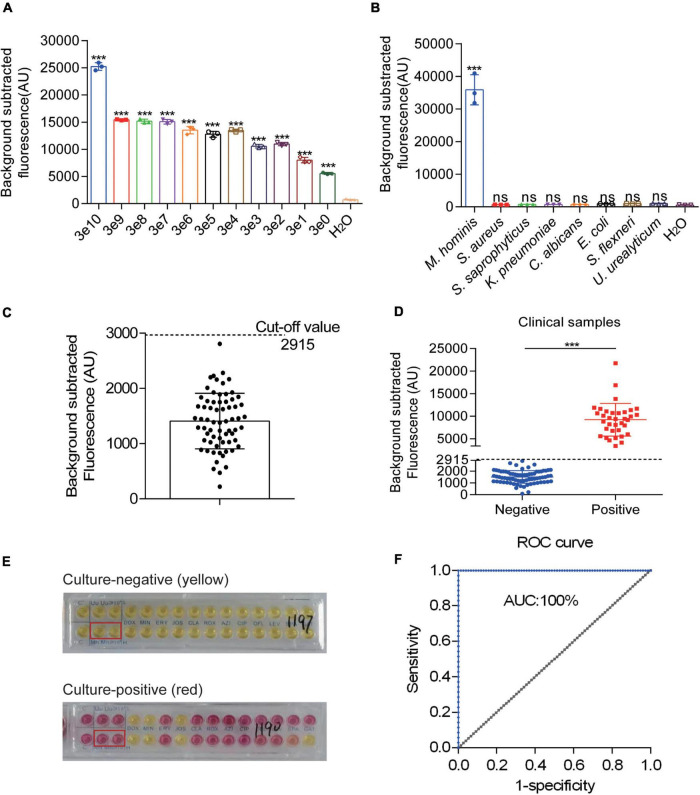
Evaluation and clinical assessment of RPA-CRISPR-Cas12a-fluorescent assay (RPA-Cas12a-Fluo). **(A)** Fluorescent results of the pMD19-T-MH gap detected by the RPA-Cas12a-Fluo assay with different copies. **(B)** Fluorescent results of different urogenital pathogens detected by the RPA-Cas12a-Fluo assay. **(C)** Fluorescent results of 67 clinical *M. hominis-*negative specimens detected by the RPA-Cas12a-Fluo assay. **(D)** Fluorescent results of 111 clinical samples detected by the RPA-Cas12a-Fluo assay. **(E)** Liquid medium culture results of negative and positive samples. **(F)** ROC curve of RPA-Cas12a-Fluo assay for detecting *M. hominis* when taking the liquid culture results as the standard. The data are presented as the mean ± SD from three independent experiments. ***p < 0.001. gap, glyceraldehyde-3-phosphate dehydrogenase; S, sample; T line, test line; C line, control line; ROC, receiver operating characteristic curve; AUC, area under the curve.

After successfully constructing a *M. hominis* detection system based on CRISPR-Cas12a, we used the pMD19-T-MH-gap to determine the LOD and specificity. Serial 10-fold dilutions of the pMD19-T-MH gap were amplified with RPA and detected by fluorescence readout. Compared with the control group, even three copies could be detected and the whole reaction only takes less than 1 h (Figure 4A). To demonstrate the specificity, other pathogenic microorganisms were tested with the RPA-Cas12a-Fluo assay. The fluorescence intensity of *M. hominis* was higher than other bacteria and pathogens including *S. aureus, S. saprophyticus, K. pneumoniae, C. albicans, E. coli, S. flexneri, U. urealyticum, C. trachomatis, N. gonorrhoeae*, and *M. genitalium* (Figure 4B and Supplementary Figure 4B). To further investigate the influence of background human genomic DNA in the RPA-Cas12a Fluo assay, the total DNA of blood and the pMD19-T-MH gap was added to the detection system. The results showed that the background human DNA has no interference with the detection (Supplementary Figure 5).

To detect *M. hominis* from the clinical samples in the RPA-Cas12a-Fluo assay, the entire detection process was completed within 60 min and the fluorescent signal was recorded. Subsequently, 67 *M. hominis*-negative samples were assayed by RPA-Cas12a-Fluo to determine the cutoff value, which turned out to be 2,915 (Figure 4C). Samples with fluorescence values higher than 2,915 were defined as *M. hominis*-positive while those with fluorescence values less than 2,915 were defined as *M. hominis*-negative. To validate the developed assay, a total of 111 clinical swab samples were tested by RPA-Cas12a-Fluo, and the results were compared with the results of liquid medium culture, which is considered as the gold standard. As shown in Figure 4D, 35 (31.53%) samples were positive and 76 (68.47%) samples were negative. Samples in liquid medium that turned from yellow to reddish or pink were defined as *M. hominis*-positive, whereas the yellow samples were defined as *M. hominis*-negative (Figure 4E). Of the samples evaluated by the liquid culture method, 36 were *M. hominis*-positive and 75 were *M. hominis*-positive (Table 1). Comparing the results of the above two detection methods, the consistency was great with Kappa = 1.000 (*p* < 0.001). The sensitivity and specificity were both 1.000 (95% CI: 0.953 to 1.000, *p* < 0.001) and the area under the curve (AUC) was 1.000 (Figure 4F). Taken together, the results indicated that the performance of the RPA-Cas12a-Fluo assay was comparable to liquid medium culture.

**TABLE 1:** The comparison of the RPA-Cas12a-Fluo assay and liquid medium culture for *M. hominis* detection in clinical samples.

RPA-Cas12a-Fluo assay		Liquid medium culture		
		Positive	Negative	Total
	Positive	35	0	35
	Negative	0	76	76
	Total	35	76	111

*RPA, recombinase polymerase amplification; Fluo, fluorescence.*

### Construction of RPA-Cas12a-LFS Assay and Validation of Clinical Samples

**FIGURE 5:**
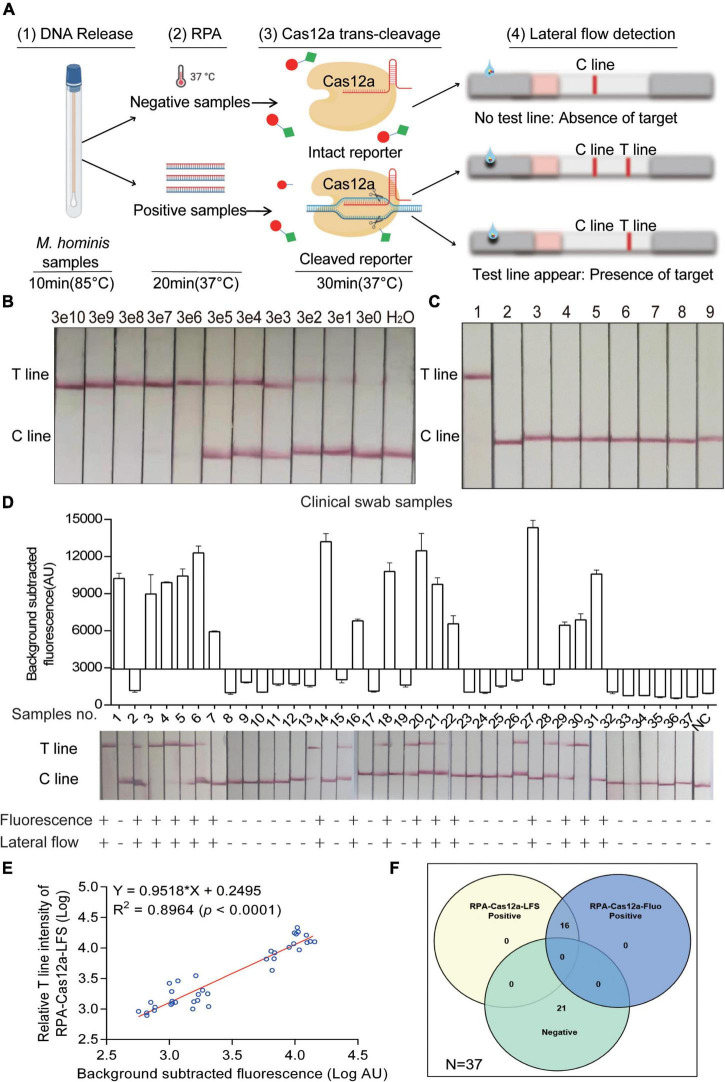
Evaluation and clinical assessment of RPA-CRISPR-Cas12a-lateral flow strip assay (RPA-Cas12a-LFS). **(A)** Schematic of the RPA-Cas12a-LFS assay for *M. hominis* detection. (1) DNA was released from clinical samples by rapid lysis for 10 min. (2) RPA amplification for 20 min. (3) Cas12a reaction with the RPA product. (4) Cas12a reaction mixture was loaded onto LFS. T line will appear with the presence of target while C line appears with the absence of target. **(B)** Lateral flow assay result of the pMD19-T-MH gap detected by the RPA-Cas12a-LFS assay with different copies. **(C)** Lateral flow assay result of different urogenital pathogens detected by the RPA-Cas12a-LFS assay. 1, *M. hominis*; 2, *C. albicans*; 3, *E. coli*; 4, *K. pneumoniae*; 5, *S. aureus*; 6, *S. flexneri*; 7, *S. saprophyticus*; 8, *U. urealyticum*. 9, H_2_O. **(D)** RPA-Cas12a-LFS results of 37 clinical samples were compared to RPA-Cas12a-Fluo. **(E)** The correlation analysis of *M. hominis* detection results generated by RPA-Cas12a-Fluo assay and RPA-Cas12a-LFS assay. The relative T line intensity was scanned and measured by ImageJ. Both the fluorescence value and the relative T line intensity were logarithmically transformed. **(F)** The Venn diagram shows the consistency between the RPA-Cas12a-Fluo assay and the RPA-Cas12a-LFS assay. The data are presented as the mean ± SD from three independent experiments. RPA, recombinase polymerase amplification; T line, test line; C line, control line.

More importantly, in order to broaden the application of the Cas12a system, we performed this system with commercial LFS to realize the visual interpretation of results in *M. hominis* nucleic acid detection. The entire detection process can be completed within 1 h, including 10 min for releasing sample genomic DNA, 20 min for RPA replication, and 30 min for the CRISPR-Cas12a reaction and readout. According to the rationale of LFS, a red T line indicates a positive sample, while the absence of a red T line indicates a negative sample (Figure 5A). We first assessed the sensitivity and specificity of the RPA-Cas12a-LFS assay. In Figure 5B, serial 10-fold dilutions of the pMD19-T-MH gap were amplified with RPA and detected by the RPA-Cas12a-LFS assay. Ultimately, the LOD also reached three copies with a visible red T line. The specificity of RPA-Cas12a-LFS assay was then tested from *M. hominis* and other urogenital bacteria including *C. albicans, E. coli, K. pneumoniae, S. aureus, S. flexneri, S. saprophyticus*, *U. urealyticum, C. trachomatis, N. gonorrhoeae*, and *M. genitalium*. The T line only appeared under the pathogen of *M. hominis*, while this line could not be detected in samples of other pathogens analyzed (Figure 5C and Supplementary Figure 4C).

Finally, to assess the efficacy of the RPA-Cas12a-LFS assay in clinical samples, 37 clinical specimens were randomly selected from 111 clinical specimens described above. These 37 clinical samples were both detected with RPA-Cas12a-Fluo and RPA-Cas12a-LFS assay. Strikingly, the results of the RPA-Cas12a-LFS assay were 100% consistent with the RPA-Cas12a-Fluo assay (Figure 5D). We further analyzed the correlation of the logarithmically transformed relative T line intensity and fluorescence value of clinical samples and found that the *R*^2^ was 0.8964 (Figure 5E). Furthermore, the relation of the above two methods was shown in a Venn diagram (Figure 5F).

## Discussion

The CRISPR system is an acquired immune defense system against nucleic acids in bacteria that includes many different Cas proteins with nuclease activity (Gootenberg et al., 2018). Among them, Cas12a has nuclease activity against DNA. Cas12a has the ability of gene editing due to its role of cleavage and collateral cleavage (Nguyen et al., 2020). Hence, it has been potentially employed for the nucleic acid detection of pathogenic microorganisms (Wang R. et al., 2021). Kanitchinda et al. (2020) combined RPA and Cas12a fluorescence assay to detect *Enterocytozoon hepatopenaei*. Compared with other Cas proteins that recognize and cleave RNA sequences such as Cas13a and Cas13b (Mustafa and Makhawi, 2021), Cas12a specifically recognizes and cleaves DNA, which is time-saving by omitting transcription in constructing the nucleic acid detection system (Yin et al., 2020). At the same time, without transcription reagents, the components of the Cas12a reaction system are simpler, which tends to be more accurate. In this study, we combined Cas12a and RPA technology to establish *M. hominis* rapid nucleic acid detection, which only takes less than 1 h.

RPA-Cas12a assay developed in this study had no cross-reactivity with other urogenital pathogens and could consistently detect three copies of DNA per reaction. In addition, when we adopted RPA for specificity test, non-specific bands appeared in the results of nucleic acid electrophoresis. Since those bands were unexpected, we were worried that those bands will affect the subsequent detection at the beginning. Surprisingly, they did not affect our results in the subsequent experiment. Non-specific bands sometimes appear in high-density samples (Liljander et al., 2009), whereas, in our study, they did not interfere with the results of the Cas12a assay. This shows that the RPA primer we designed is specific and the established detection system is also available. Also, it is worth mentioning that *M. hominis* and *M. genitalium* have high homology, especially the information of gap gene (Baczynska et al., 2004), making it difficult to distinguish the two pathogens in clinical detection. This Cas12a detection system established in this study can accurately distinguish *M. hominis* from *M. genitalium.* Therefore, it is of great significance for clinical detection.

Since the urinary tract swabs usually contain limited amount of bacteria, if it is extracted by boiling (Andreou, 2013) simply, a lot of nucleic acid may be lost, which makes it hard to estimate and detect the nucleic acid from *M. hominis*. In order to ensure the amount of nucleic acid extracted and speed up the whole detection process, we used the direct lysis buffer with a fast nucleic acid extraction kit, which can quickly extract adequate nucleic acid within 10 min. Additionally, we optimized the components of the reaction system including the concentration of RPA primers, the selection of crRNA, and the ratio of Cas12a protein to crRNA. All of the efforts ensured that the whole reaction process can be completed within 1 h.

Although the Cas12a detection system we built can be readily operated, considering its dependence on a fluorescence detective instrument that represents a barrier to the implementation of portable devices for *M. hominis* diagnostics, we further applied commercial LFS to achieve the visual interpretation of results. The experimental results showed that the LFS detection preserved the high sensitivity of the RPA-Cas12a reaction with an LOD of three copies, and the specificity is also considerable. So far, we have established the RPA-Cas12a-LFS assay, which may be used as an auxiliary means of nucleic acid detection in clinical practice in the near future.

Thus far, several detection platforms have been developed for *M. hominis*, including liquid medium culture, qRT-PCR, and ELISA. In Table 2, we have listed several methods to detect *M. hominis.* Although these methods offer good sensitivity, they are limited by various constraints such as being time-consuming and lack of compatibility with point-of-need applications. For example, in spite of liquid medium culture being determined as the gold standard, it is still slow and laborious (de Souza et al., 2009). As to qRT-PCR or PCR detecting *M. hominis* nucleic acid, it still needs several large laboratory instruments and, hence, more practical for laboratory settings than for field work (Yang et al., 2021). Although ELISA can also be used to determine the antigen of *M. hominis* (Miettinen et al., 1984), it is still necessary to detect nucleic acid to determine the infection of *M. hominis.* However, the CRISPR-Cas12a detection system takes less time and is more convenient for rapid screening. Also, once the sample is confirmed to be positive, the drug sensitivity test with liquid medium culture can be carried out, which is more cost-saving.

**TABLE 2:** Comparison of different detection methods for *M. hominis*.

Method	Time	Assay type	Main instrument	LOD	Strengths	Weakness	References
Liquid Medium Culture	48–72 h	Metabolic	Incubator	10,000 copies/μl	Simultaneous drug resistance tests	Time-consuming, false-positive risk	Keskin et al., 2018
qRT-PCR	2–4 h	Nucleic acid	Real-time qRT-PCR instrument	7 copies/μl	High specificity and sensitivity; quantitative detection	Complex operations, expensive cost	Ferandon et al., 2011
ELISA	2–4 h	Antigen and antibody	Microplate reader	10 ng/ml	High specificity	Complex operations, false-positive risk	Miettinen et al., 1984
RPA-Cas12a-Fluo	1 h	Nucleic acid	Fluorescence microplate reader	3 copies/μl	High specificity and sensitivity	Qualitative testing	This study
RPA-Cas12a-LFS	1 h	Nucleic acid	(Naked eyes)	3 copies/μl	High specificity and sensitivity; point-of-care testing	Qualitative testing	This study

*LOD, limit of detection; qRT-PCR, quantitative real-time polymerase chain reaction; ELISA, enzyme-linked immunosorbent serologic assay; RPA, recombinase polymerase amplification; Fluo, fluorescence; LFS, lateral flow strip.*

Additionally, *U. urealyticum* usually causes urogenital tract infection, and it is often accompanied by *M. hominis* infection (Jamalizadeh et al., 2014; Lee et al., 2016; Moragianni et al., 2019). Therefore, we need to further improve the RPA-Cas12a-LFS detection system to detect *M. hominis* and *U. urealyticum* infection, such as developing a multi-detective platform combined with other Cas proteins, which is more meaningful for clinical practice.

Although the LFS with gold nanoparticles conjugated anti-FITC is very convenient to use, it is difficult to perfectly prepare gold nanoparticles with uniform size and single dispersion. Heterogeneous gold nanoparticles are difficult to uniformly coat the corresponding antibodies, which makes them prone to non-specific aggregation, and their own scattered light interference will also bring a high background signal (Bamrungsap et al., 2012). Therefore, in order to solve the problem, new materials such as quantum dots and polypropylene microspheres (Chen et al., 2017, 2020) need to be introduced to realize the qualitative and quantitative interpretation of the RPA-Cas12a-LFS system. Taken together, we have attempted to establish an *M. hominis* RPA-Cas12a-LFS detection system with two output modes, which were fluorescence signal and strips. These two methods complement each other and greatly improve the detective accuracy, which are more persuasive and reliable for evaluating *M. hominis* infection and expected to be applied in clinical practice.

## Conclusion

In this study, we demonstrated a novel detection method for the diagnosis of *M. hominis* infection with high sensitivity and specificity based on the RPA-CRISPR-Cas12a detection. The developed detection procedure requires minimal template with an LOD of 3 *M. hominis* copies, little amount of reagents, and a convenient reaction condition, and is time-saving, which meets the needs of clinical detection of *M. hominis*. The method did not exhibit false-positive results with other urogenital pathogens, and was robust to the presence of abundant background DNA from human. Also, we attempted to combine LFS with the RPA-CRISPR-Cas12a detection to get rid of the dependence on large instruments. Therefore, the RPA-Cas12a detection shows great promise for routine detection of *M. hominis*, and is highly accessible to population screening or resource-limited settings in molecular diagnostics.

## Data Availability Statement

The original contributions presented in the study are included in the article/Supplementary Material, further inquiries can be directed to the corresponding author/s.

## Ethics Statement

The studies involving human participants were reviewed and approved by Medical Ethics committee of Shantou Second People’s Hospital. The patients/participants provided their written informed consent to participate in this study.

## Author Contributions

JC, YH, and BX designed and performed the experiments. HD collected the clinical specimens. KG and KL performed the data analysis. WH wrote the manuscript. WH and LL conceived and designed the project. All authors contributed to the article and approved the submitted version.

## Conflict of Interest

The authors declare that the research was conducted in the absence of any commercial or financial relationships that could be construed as a potential conflict of interest.

## Publisher’s Note

All claims expressed in this article are solely those of the authors and do not necessarily represent those of their affiliated organizations, or those of the publisher, the editors and the reviewers. Any product that may be evaluated in this article, or claim that may be made by its manufacturer, is not guaranteed or endorsed by the publisher.

## Footnotes

1
https://www.ncbi.nlm.nih.gov/tools/primer-blast/index.cgi?LINK_LOC=BlastHome

2
http://bioinfolab.miamioh.edu/CRISPR-DT/interface/Cpf1_design.php

## Abbreviations

qRT-PCR: quantitative real-time polymerase chain reaction
gap: glyceraldehyde-3-phosphate dehydrogenase
CRISPR: clustered regularly interspaced short palindromic repeats
IPTG: isopropyl-1-thio- β -D-galactopyranoside
RPA: recombinase polymerase amplification.
