# Supplementary material for: Development of a RPA-CRISPR-Cas12a Assay for Rapid, Simple, and Sensitive Detection of Mycoplasma hominis
Source: Front Microbiol. 2022 Apr 8;13:842415. doi: 10.3389/fmicb.2022.842415 (PMC9024404; doi:10.3389/fmicb.2022.842415)
Supplement: Supplementary file 1 [file Data_Sheet_1.docx]

***Supplementary Material***


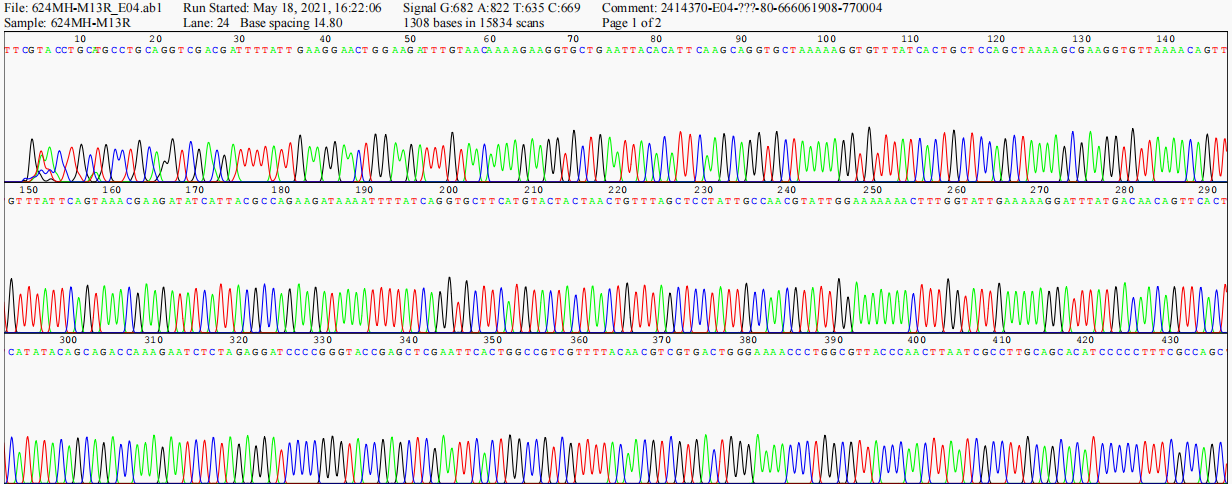


**SupplementaryFigure 1.** Sequencing results of the recombinant plasmid pMD19-T-MH gap (partial). A 280bp amplicon were determined by the forward primer (5′-TTATTGAAGGAACTGGAAGATTTGTA-3′) and reverse primer (5′-CTTTGGTCTGCTGTATATGAGTGAAC-3′).


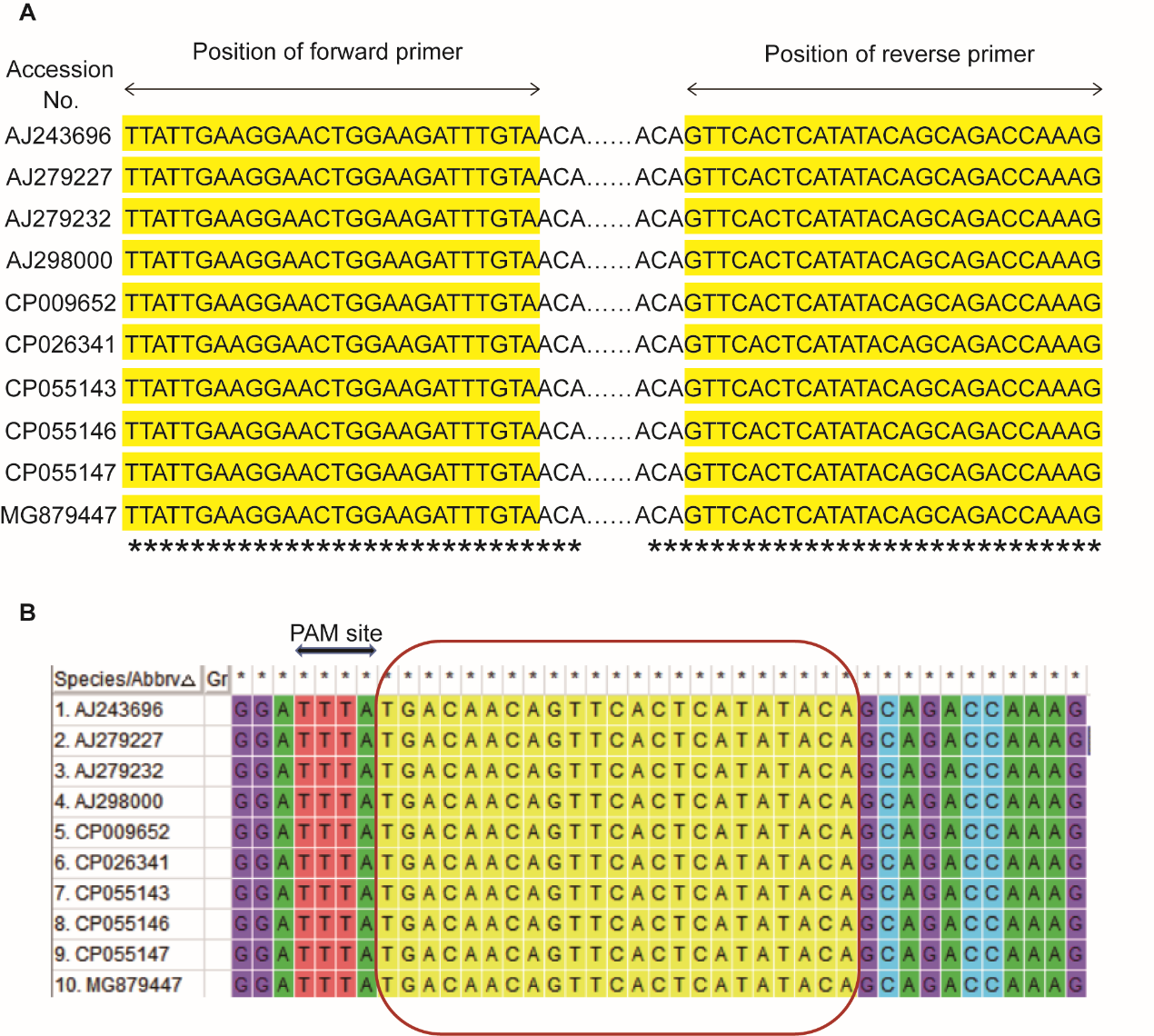


**Supplementary Figure 2.**Multiple sequence alignment of *M. hominis* gap gene. (A) The gap sequences of 10 *M. hominis*isolates were downloaded from NCBI and compared by MEGA7. Highlighted are the primer sequences. *The matching bases. (B) The conserved sequence was selected as the best target sequence of crRNA (crRNA 3).Highlighted are the best target sequence of crRNA sequences. *The matching bases.





**Supplementary Figure 3.**Fluorescent results of the pMD19-T-MH gap plasmid detected by the RPA-Cas12a-Fluo assay with different Cas12a.

The data are presented as the mean ± SD from three independent experiments. **, *p<*0.001.

Abbreviations: gap, glyceraldehyde-3-phosphate dehydrogenase;MH, *M. hominis*; ns, not significant.


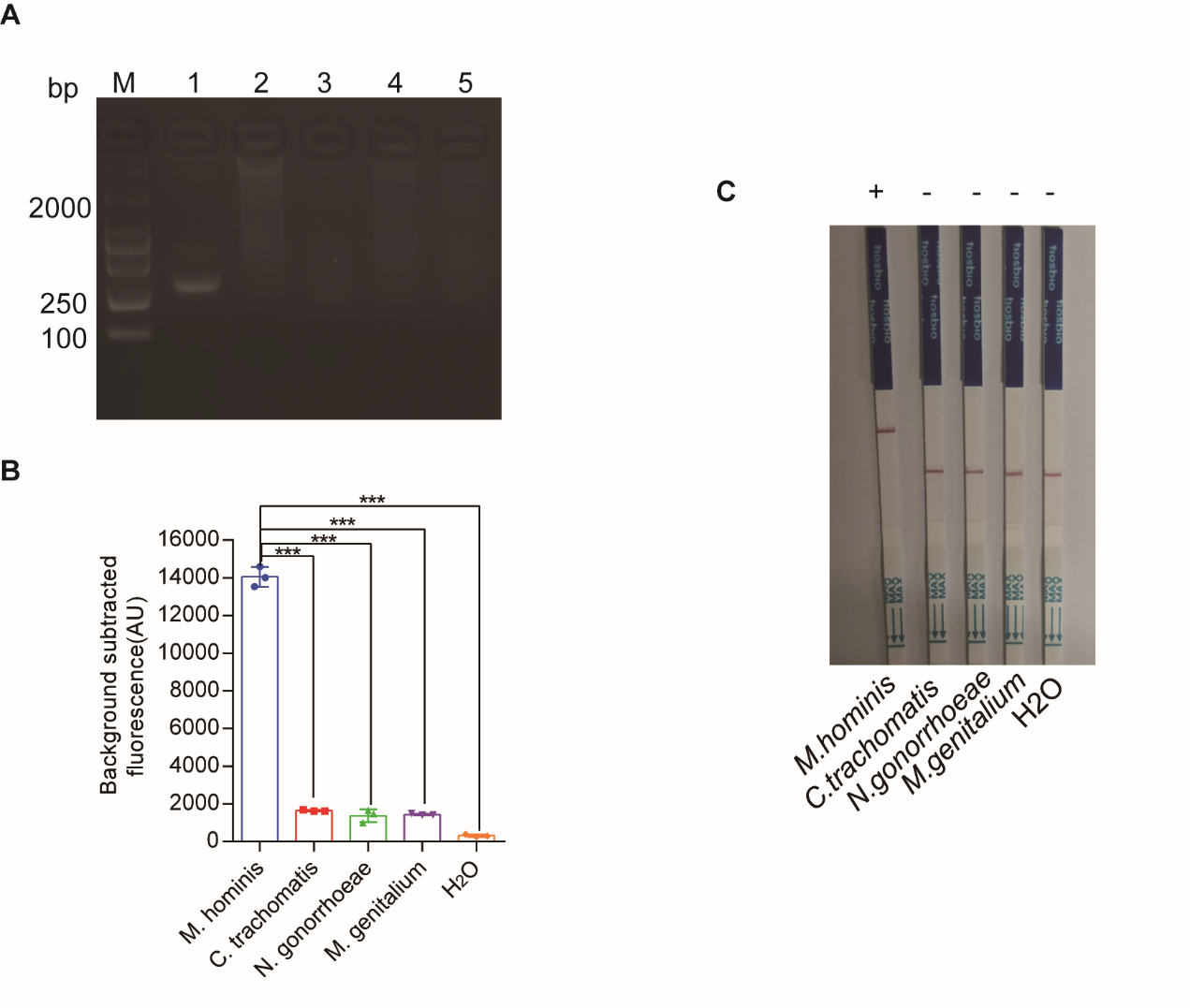


**Supplementary Figure 4.** Specificity of RPA-CRISPR-Cas12a assayfor detection of *M. hominis*.

1. Agarose gel electrophoresis for detecting RPA products with other different urogenital pathogens. 1, *M. hominis*;2,*C. trachomatis; 3, N. gonorrhoeae; 4, M. genitalium;* 5, H_2_O.
2. Fluorescent results of different urogenital pathogens detected by the RPA-Cas12a-Fluo assay.
3. Lateral flow assay result of different urogenital pathogens detected by the RPA-Cas12a-LFS assay.

The data are presented as the mean ± SD from three independent experiments. ***, *p<*0.0001.

Abbreviations: bp, base pair; M, marker; gap, glyceraldehyde-3-phosphate dehydrogenase.





**Supplementary Figure 5.**The effect of background human DNA onthe RPA-Cas12a-Fluo assay.

The data are presented as the mean ± SD from three independent experiments.

Abbreviations: gap, glyceraldehyde-3-phosphate dehydrogenase; MH, *M. hominis*; ns, not significant.

**Supplementary Table 1.**Information on purchased reagents

| Name | Company and item number |
| --- | --- |
| HiScribe™ T7 Quick High Yield RNA Synthesis Kit | NEB, E2050S |
| 6His-MBP-TEV-huAsCpf1 plasmid | MiaoLingBio, P5327 |
| DL2000,5000 DNA Marker | Takara3580Q |
| Total RNA Cleanup Kit | Foregene, RE-03014 |
| Premix Taq | Takara, R004A |
| PMD^TM^ 19T Vector cloning Kit | Solarbio, AK91497A |
| Fluorescent reporter and biotin reporter, | Biolifesci,M20802-P001A |
| KOD DNA polymerase（UKOD）2× Master Mix | Toyobo,KMM-101 |
| Rosetta 2 (DE3) pLysS | TOLOBIO, CC96110-01 |
| Ni NTA Beads 6FF | Smart-lifesciences, SA005010 |
| TIANamp Genomic DNA Kit | Tiangen, DP304-02 |
| Chimney 384 wells Plates (black) | Greiner Bio, 781076 |

**Supplementary Table 2.**Sequences involved in this study

| Name | Sequence (5′-3′) |
| --- | --- |
| Target dsDNA MH gap gene region | TTATTGAAGGAACTGGAAGATTTGTAACAAAAGAAGGTGCTGAATTACACATTCAAGCTGGTGCTAAAAAGGTATTTATCACTGCTCCAGCTAAAAGCGAAGGCGTTAAAACAGTTGTTTATTCAGTAAACGAAGATATAATTACACCAGAAGATAAAATTTTATCAGGCGCTTCATGTACTACTAACTGTTTAGCTCCTATTGCCAACGTATTGGAAAAAAACTTTGGTATTGAAAAAGGATTTATGACAACAGTTCACTCATATACAGCAGACCAAAG |
| Target dsDNA MH merase coding region (ATCC 27545) | TTCGTACCTGCATGCCTGCAGGTCGACGATTTTATTGAAGGAACTGGAAGATTTGTAACAAAAGAAGGTGCTGAATTACACATTCAAGCAGGTGCTAAAAAGGTGTTTATCACTGCTCCAGCTAAAAGCGAAGGTGTTAAAACAGTTGTTTATTCAGTAAACGAAGATATCATTACGCCAGAAGATAAAATTTTATCAGGTGCTTCATGTACTACTAACTGTTTAGCTCCTATTGCCAACGTATTGGAAAAAAACTTTGGTATTGAAAAAGGATTTATGACAACAGTTCACTCATATACAGCAGACCAAAGAATCTCTAGAGGATCCCCGGGTACCGAGCTCGAATTCACTGGCCGTCGTTTTACAACGTCGTGACTGGGAAAACCCTGGCGTTACCCAACTTAATCGCCTTGCAGCACATCCCCCTTTCGCCAGCTGGCGTAATAGCGAAGAGGCCCGCACCGATCGCCCTTCCCAACAGTTGCGCAGCCTGAATGGCGAATGGCGCCTGATGCGGTATTTTCTCCTTACGCATCTGTGCGGTATTTCACACCGCATATGGTGCACTCTCAGTACAATCTGCTCTGATGCCGCATAGTTAAGCCAGCCCCGACACCCGCCAACACCCGCTGACGCGCCCTGACGGGCTTGTCTGCTCCCGGCATCCGCTTACAGACAAGCTGTGACCGTCTCCGGGAGCTGCATGTGTCAGAGGTTTTCACCGTCATCACCGAAACGCGCGAGACGAAAGGGCCTCGTGATACGCCTATTTTTATAGGTTAATGTCATGATAATAATGGTTTCTTAGACGTCAGGTGGCACTTTTCGGGGAAATGTGCGCGGAACCCCTATTTGTTTATTTTTCTAAATACATTCAAATATGTATCCGCTCATGAGACAATAACCCTGATAAATGCTTCAATAATATTGAAAAAGGAAGAATATGAGTATTCAACTTTCCGGGTCGCCTTATTCCCTTTTTGCGGATTTTGCCTCCTGTTTTGCTCCCCAGAACCCTGGGAAATTAAAAAGCTGAAATCATTGGGGGCCAAGGGGTTACTCAAATGGTTCCCCGGGGAAAATCTTGAAAATTTCCCCGAAAAATTTCCCAGGAACTTTAATTTTTGTTGGGGGGGGTATTCCGTTGTCGCGGGGAAAACCGGCCCACATTTTCCAAAATGGGGGACCCCCCACAAAATCTTGGGGTGGAAAAAAAATTGGCCCCCACAGAACCCCCTCCCCCCCACAAAGAAACTCTTTTGGGGGTTTGCGAAAACACCCCAACCAACAAAAACATTTAGAGAAGAG |

crRNAs

| crRNA | Sequences (5′-3′) |
| --- | --- |
| crRNA 1 | UAAUUUCUACUCUUGUAGAUUCACUGCUCCAGCUAAAAGCGAA |
| crRNA 2 | UAAUUUCUACUCUUGUAGAUUCAGGCGCUUCAUGUACUACUAA |
| crRNA 3 | UAAUUUGUACUCUUGUAGAUUGACAACAGUUCACUCAUAUACA |
| False crRNA | UAAUUUCUACUCUUGUAGAUUAGAUACCUGUGUAGUCAAGAAU |
| T7 primer | TAATACGACTCACTATAGGG |

RPA primers

| Primer name | Sequences (5′-3′) |
| --- | --- |
| MH16srRNA-1Forward | TGATGTTTAGCCGGGTCGAGAGACTG |
| MH16srRNA-1Reverse | TCCGAAGACCTTCATCGTGCACGCTG |
| **MH gap-2Forward** | **TTATTGAAGGAACTGGAAGATTTGTA** |
| **MH gap-2Reverse** | **CTTTGGTCTGCTGTATATGAGTGAAC** |
| MH16srRNA-3Forward | GTTTAGCCGGGTCGAGAGACTGAACG |
| MH16srRNA-3Reverse | GTCGCTCCATCAAGCTTTCGCTCATTG |

ssDNA reporter

| Primer name | Sequences (5′-3′) |
| --- | --- |
| ssDNA reporter-Fluo | 5′-/6-FAM/TTTTTT/BHQ1/-3′ |
| ssDNA reporter-LFS | 5′-/6-FITC/TTTTTTT/Biotin/-3′ |

**Supplementary Table 3.**Frist, add to the PCR tube according to the system:

| Component | Volume to add (μL) |
| --- | --- |
| Premix Taq buffer | 25 |
| Sample DNA | 10 |
| MH gap-2Forward,10μM | 0.3 |
| MH gap-2Reverse,10μM | 0.3 |
| Add water to total | Up to 50 |

The amplified PCR product has an "A" base attached to its 3 'end. The product can be directly cloned into pMD 19T-vector.

I In vitro transcription (IVT) steps of crRNA

Following the annealing of the transcription templates with T7 promoter primer, hybridized DNA oligonucleotides were extended to form double-stranded transcription templates.

**Supplementary Table 4.** The above products were mixed in the following system:

| Component | Volume to add (μL) |
| --- | --- |
| Nuclease free water | 12 |
| crRNA template,100μM | 4 |
| T7 primer,100μM | 2 |
| PCR buffer | 2 |
| Total volume | 20 |

Next, anneal the PCR tube by performing denaturation at 95°C 5min and 55 °C 30s. The reaction was then slowly cooled to 4°C in a PCR thermocycler.

II T7 transcription of crRNA

**Supplementary Table 5.** The above products were mixed in the following system:

| Component | Volume to add (μL) |
| --- | --- |
| Nuclease free water | 34 |
| NTP buffer Mix | 20 |
| T7 RNA polymerase mix | 4 |
| Annealing reaction | 20 |
| Total volume | 78 |

The HiScribe™ T7 Quick High Yield RNA Synthesis Kit (NEB, E2050S) was mixed according to the above system and placed in a 37°C incubator for 16 hours. Then the reaction was slowly cooled to 4°C in a PCR thermocycler.

III Purification of crRNA

The above T7 transcript product was purified by using the Total RNA Cleanup Kit (Foregene, RE-03014) and following the instructions strictly.

**Supplementary Table 6.** Reaction system of RPA.

| Component | Volume to add (μL) |
| --- | --- |
| Primer Free Rehydration buffer | 29.5 |
| Forward Primer (10μM) | 2.4 |
| Reverse Primer (10μM) | 2.4 |
| Template and water to | 13.2 |
| 280nM Magnesium Acetate (MgOAc) | 2.5 |
| Total volume | 50 |

The TwistAmp® basic kit (TwistDx Inc., Cambridge, UK) was mixed according to the above system and placed in a 39°C incubator for 20min, after the reaction is finished, store on ice.

**Supplementary Table 7.** Reaction system of RPA-CRISPR-Cas12a.

| Component | Volume to add (μL) |
| --- | --- |
| RPA sample (10-fold dilutions) | 2 |
| crRNA (62.5nM) | 1 |
| AsCas12a (250nM) | 1 |
| ssDNA FQ reporter (25pmol/μL) | 1 |
| 10x reaction buffer | 2 |
| Add water to total | 20 |

The fluorescence assay was performed by mixing the above system and then performing a fluorescence collection assay. However, the lateral flow test strip needs to be incubated for 30 min at 37°C in a warm metal bath, and then 80μL PBS buffer is pipetted and mixed to reach the required volume of the lateral flow test strip. After 3 minutes, the results can be visible to the naked eye.
